# Un savon pour lutter contre les schistosomoses : une intervention de terrain à reprendre ?

**DOI:** 10.48327/mtsi.v4i2.2024.508

**Published:** 2024-04-08

**Authors:** Jean-Loup REY

**Affiliations:** Groupe d’intervention en santé publique et épidémiologie (GISPE)

**Keywords:** Schistosomose, Lutte, Bétaïnes, Savon, Niger, Côte d’Ivoire, Afrique subsaharienne, Schistosomiasis, Control, Betaine, Soap, Niger, Côte d’Ivoire, Sub-Saharan Africa

## Abstract

Une expérience a été menée en 1985-87 contre les schistosomoses par l’utilisation de produits neutralisant les stades intermédiaires des schistosomes. Au laboratoire, il avait été montré que les lauryl-bétaïnes, substances amphotères, utilisées pour les shampoings pour enfants, immobilisaient rapidement miracidiums et cercaires. Des études au Niger dans les conditions de terrain avec des eaux chargées en matières organiques ont donné des résultats similaires. Cet agent de surface peut être incorporé dans des savons ordinaires à la dose de 5 % sans modifier leurs caractéristiques. Des savons bétaïnés ont été mis en vente dans les circuits commerciaux ordinaires au Niger puis en Côte d’Ivoire, dans des villages hyperendémiques pour *Schistosoma haematobium.* Les bétaïnes ont diffusé dans les eaux utilisées par les populations pour se laver sans intervention extérieure. Ces savons ont été bien acceptés par ces populations. Après un an, le résultat par rapport à des villages témoins est mitigé sur la dynamique de la schistosomose urinaire en termes de prévalence et d’oviurie. Un traitement anti-schistosomes semble nécessaire au début de l’intervention. L’utilisation des savons par les populations est à mesurer. En conclusion, cette action prometteuse en laboratoire mériterait d’être à nouveau évaluée sur le terrain, en complément des actions de sensibilisation et de traitement systématique.

## Abstract (en)

An experiment was carried out in 1985-87 against schistosomiasis using products neutralizing the intermediate stages of schistosomes. In the laboratory, it had been shown that lauryl betaines, amphoteric substances, used for children's shampoos, quickly immobilized miracidiums and cercariae. Studies in Niger in field conditions with water laden with organic matter gave similar results. This surfactant can be incorporated into ordinary soaps at a dose of 5% without changing their characteristics. Betaine soaps were put on sale in ordinary commercial channels in Niger then in Côte d’Ivoire, in hyperendemic villages for *Schistosoma haematobium.* Betaines diffused without external intervention into the water used by populations for washing. The soaps were well accepted by these populations. However, after one year, the results in tested villages compared to control ones were unclear on the dynamics of urinary schistosomiasis in terms of prevalence and oviuria. Anti-schistosome treatment seems necessary at the start of the procedure. The use of soap by populations needed to be measured. In conclusion, this promising laboratory action deserves to be evaluated again in the field, in addition to health education and systematic treatment actions.

Les schistosomoses ont un cycle complexe procurant plusieurs cibles d’attaque pour une lutte efficace associant plusieurs moyens. La lutte contre les mollusques hôtes intermédiaires a fait l’objet dans le passé de nombreuses études, de même que les traitements antiparasitaires à large échelle dans les communautés, notamment chez les enfants d’âges scolaires, les plus vulnérables [1]. Il a ainsi été proposé de développer l’élevage de poissons, de canards et même d’écrevisses malacophages. Parmi les stratégies de contrôle des schistosomoses, la lutte contre les stades intermédiaires fut plus rarement étudiée.

Nous rapportons ici une expérience menée dans les années 1980, au Niger puis en Côte d’Ivoire, qui mériterait d’être reconsidérée en appui aux stratégies médicamenteuses et de développement. Dans les années 1980, les essais thérapeutiques concernant le praziquantel (Biltricide®) et l’oltipraz (abandonné à cause des effets secondaires) étaient en cours, les traitements utilisés étaient le metrifonate (Bilarcil®) et le niridazole (Ambilhar®). La lutte était essentiellement basée sur l’éducation pour la santé et l’assainissement. L’utilisation des molluscides et de la lutte biologique était perçue comme difficile [4].

Grâce aux travaux de Claude Combes (19352021) à Perpignan, faisant suite à des travaux chinois, nous avons eu l’occasion de tester une méthode visant les miracidiums et surtout les furcocercaires (Fig. 1). En 1982, Combes et Arnaudis avaient testé différents produits tensio-actifs. Ils avaient montré que deux produits amphotères, des lauryl-bétaïnes, avaient une activité inhibitrice puissante sur les cercaires de schistosomes élevés au laboratoire. Ces produits constituent la base des shampoings pour bébés et enfants et n’avaient aucune toxicité humaine connue [2, 3].

**Figure 1:**
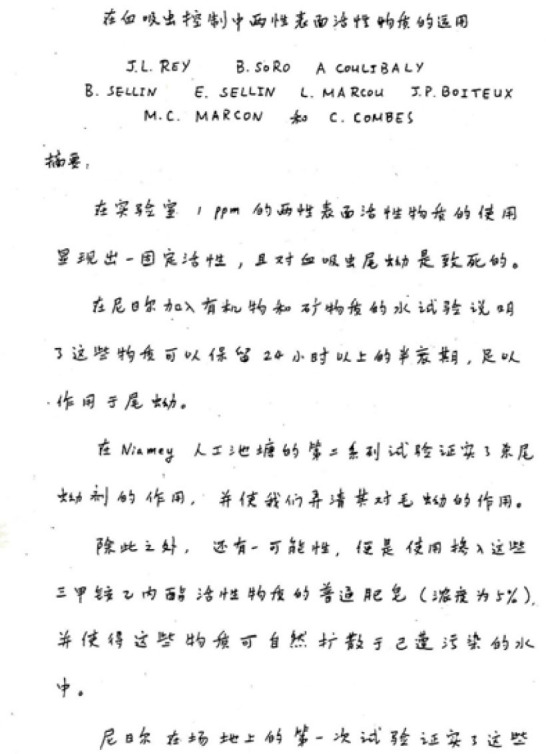
Résumé en chinois de l’étude Chinese summary of the study

Au Niger, en 1984, des essais en eaux chargées de matières organiques (mares et rizières) ont montré que ces produits gardaient une demi-vie suffisante, supérieure à 24 heures, pour une action sur les cercaires (Fig. 2 et 3). Des essais dans une mare artificielle avec remplissage par les pompiers d’eau du fleuve (Fig. 4) puis remplie par nos soins d’une eau venant des rizières (Fig. 5) confirmaient l’action inhibitrice sur des cercaires de *S. mansoni.* Dans la mare témoin, 29 % des mollusques étaient infectés contre 0 % dans la mare traitée avec 1 ppm de bétaïnes [5, 6]. Un contrôle dans une mare naturelle des environs de Niamey a montré qu’il n’y avait aucun effet nuisible sur la faune aquatique à cette dose.

**Figure 2:**
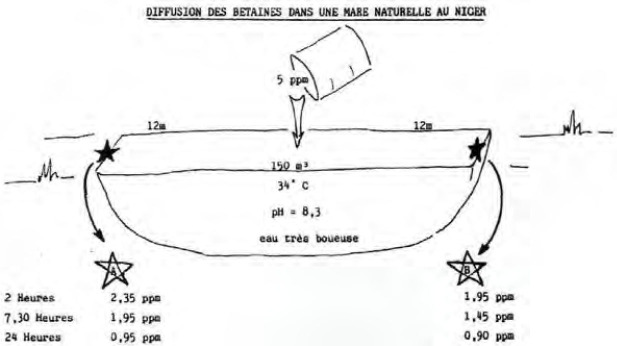
Essai dans une mare naturelle Test in a natural pond

**Figure 3:**
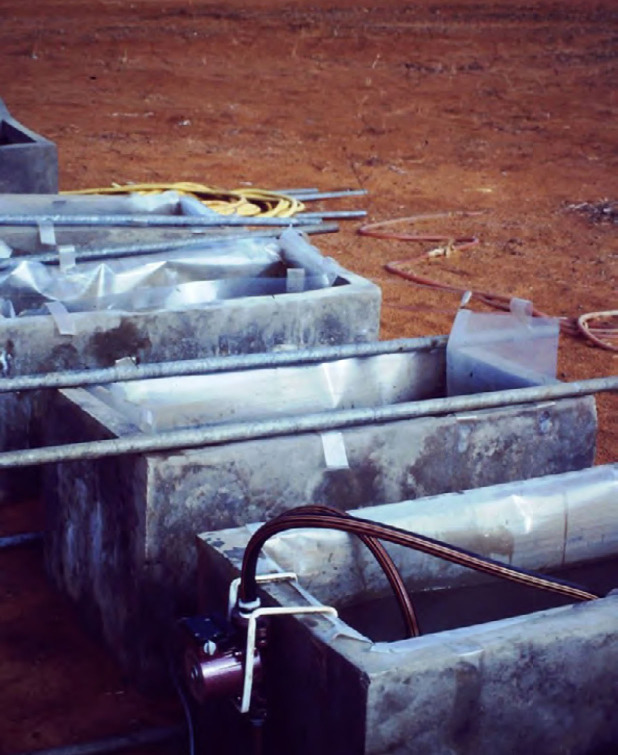
Essais avec des eaux plus ou moins chargées en matière organiques Tests with water more or less loaded with organic matter

**Figure 4:**
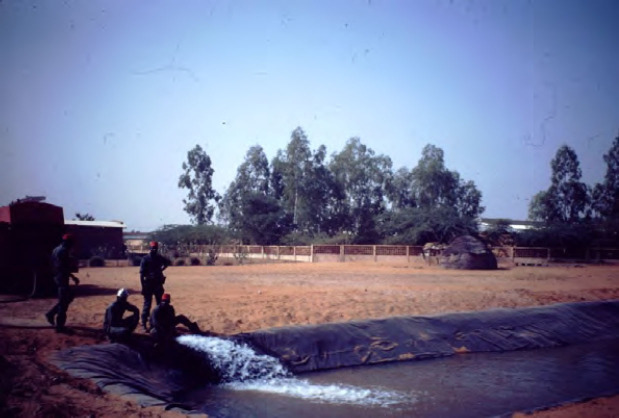
Mare artificielle créée avec l'aide des pompiers Artificial pond created with the help of the fire department

**Figure 5:**
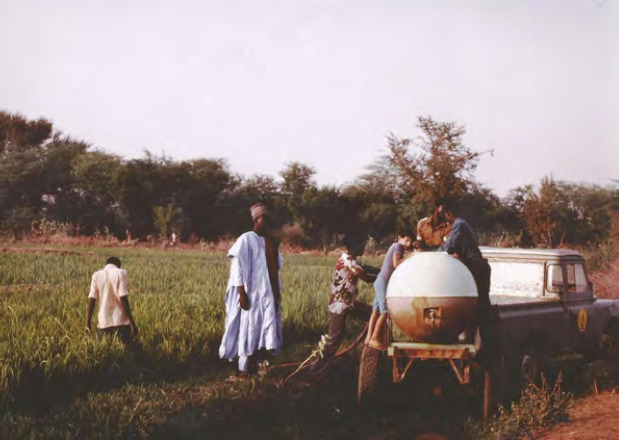
Récolte d'eau de rizières Pumping water from rice paddies

Ces bétaïnes pouvaient être incorporées dans des savons ordinaires permettant, à la concentration de 5 %, leur diffusion suffisante dans les canaux et mares. Une tonne de ces savons a été fabriquée pour être testée sur le terrain. Dans deux villages hyperendémiques pour la schistosomose urinaire de la zone rizicole proche de Niamey (Fig. 6), nous avons traité tous les habitants par trois doses de métrifonate puis mis en place dans un des deux villages les savons bétaïnés. Cette diffusion a été effectuée par l’intermédiaire des commerçants locaux. Les savons étaient vendus au même prix que les autres savons. Le fabricant nigérien de savons (Société nigérienne de produits chimiques) a distribué à tous les commerçants de la zone ce savon bétaïné marqué d’un petit signe discret au lieu du savon habituel, sans en informer les commerçants. L’accès à cette formule de savon a ainsi été mis en place sans intervention particulière.

**Figure 6:**
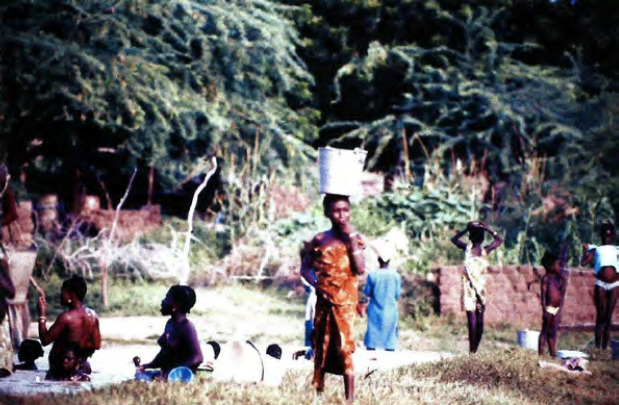
Utilisation de l'eau des rizières pour toilette et lessive Using water from rice paddies for bathing and laundry

Cependant, l’essai a été interrompu après huit mois sans montrer d’effets significatifs sur la prévalence parasitologique. Il a cependant montré que la stratégie était acceptée par la population. Il est probable qu’une réduction plus importante de la prévalence parasitaire doit être obtenue au départ pour avoir des résultats tangibles.

Un autre essai a été réalisé en 1986-87 en Côte d’Ivoire dans des conditions réelles. Tous les enfants scolarisés (classe d’âge la plus atteinte) éliminant des œufs dans deux villages endémiques pour *S. haematobium* ont été traités par praziquantel (dose unique de 40 mg/kg). Les habitants d’un des villages ont ensuite utilisé des savons avec bétaïnes. Après un an, les résultats sont résumés ainsi (Tableau I):

**Tableau I:** Comparaison des données parasitologiques de deux villages de Côte d'Ivoire Comparison of parasitological data from two villages in Côte d'Ivoire

	1986	1987
**Village témoin**		
effectif	228	376
prevalence globale	55,3 %	20,2 %
prevalence sujets traités	100 %	15,2 %
oviurie moyenne globale	42,7	10,4
oviurie sujet traités	48,3	5,1
**Village testé**		
effectif	126	177
prévalence globale	66,6 %	54,2 %
prévalence sujets traités	100 %	45,7 %
oviurie globale	121,8	70,3
oviurie sujets traités	164,1	5,9

Oviurie : nombre dœufs pour 10 ml d’urines.

Les résultats ne sont pas probants. Dans le village test, les indicateurs parasitaires sont améliorés mais pas plus que dans le village témoin. Ce bilan pourrait être dû à deux facteurs :

1. le village testé présentait une oviurie moyenne plus élevée au départ;
2. lors du contrôle à un an, 40 % des enfants scolarisés du village n’avaient pas été traités l’année précédente, car absents.

Cependant, ces essais ont montré la faisabilité du processus. Dans ces deux essais de terrain, les stocks de savons prévus selon les données de consommation antérieure ont été épuisés et aucune plainte ou question n’a été relevée.

Ce moyen de lutte a une action permanente sur le cycle, réalisée sans intervention extérieure directe. Des études complémentaires pourraient confirmer cet intérêt : dosage des bétaïnes dans l’eau des mares et marigots, place et importance du traitement antiparasitaire de départ, effets collatéraux sur la faune aquatique, etc.

Si l’efficacité est prouvée, il faudra alors examiner la place de ce moyen de lutte par rapport aux outils existants : traitement de masse (avec risque d’apparition de résistances), molluscicides, prédateurs (surtout efficaces pour *S. japonicum),* éducation pour la santé et hygiène collective, actions sur les réservoirs de mollusques. Les stratégies actuelles basées essentiellement sur le traitement de masse donnent de très bons résultats mais il faut craindre l’apparition de résistances et envisager des actions complémentaires pour un contrôle de cette maladie.

## Liens d'intérêt

L’auteur ne déclare aucun conflit d’intérêts.
